# Navigating a new home: a grounded theory model of sports as a catalyst for social integration among aging migrants in China

**DOI:** 10.3389/fpubh.2026.1731904

**Published:** 2026-05-13

**Authors:** Sun Wei, Wang Kunyan

**Affiliations:** 1 College of Business, Yancheng Teachers University, Yancheng, China; 2 Yancheng Institute of Technology, Yancheng, China

**Keywords:** aging migrants, identity recognition, lifelong wellbeing, social integration, social justice, sport social work, sports activities

## Abstract

**Introduction:**

The rapidly expanding aging migrant population in China presents a critical challenge for both public health and social justice, as poor social integration is linked to heightened risks of social isolation and mental health issues. While sports are recognized as a potential vehicle for integration, the specific mechanisms through which they can be leveraged as a professional social work intervention remain theoretically underdeveloped.

**Methods:**

This study employed a rigorous grounded theory approach, analyzing multi-source data from 34 aging migrants and 23 supplementary texts, to construct a novel multidimensional model.

**Results:**

Our findings reveal a dynamic system composed of six core elements: social inclusiveness (external conditional system) and intergenerational relationships (internal kinetic mechanism) create the foundational capacity for participation; social networks (behavioral-structural driver) and social memory (behavioral-attributive driver) operate synergistically to execute the integration process; and psychological integration and identity recognition mark the successful internalization of belonging. Crucially, these elements form a dynamic cycle, offering a roadmap for intervention.

**Discussion:**

This model moves beyond fragmented indicators to provide a holistic theoretical explanation. Crucially, it extends the value of sports beyond the playing field, offering a practical framework for sport social workers to foster inclusive communities. By elucidating the roles of intergenerational dynamics and social memory, the research provides actionable insights for advancing the social rights and lifelong wellbeing of this vulnerable demographic.

## 1 Introduction

In China, population migration is increasingly characterized by family-oriented movement. Within this trend, aging migrants represent a rapidly expanding “silver wave,” fundamentally reshaping traditional migration patterns. Current statistics estimate 18 million aging migrants in China, constituting 7.2% of the total national mobile population of 247 million. Notably, 43.0% relocate specifically to support their adult children’s families (1). Driven by traditional family values, this number is projected to rise. However, deep-seated nostalgia, insufficient policy support, and inadequate social security measures often hinder their integration into host cities (2). As their numbers swell, the social integration of aging migrants has evolved from a familial concern into a critical public health challenge with implications for global strategies on population aging. Poor social integration is closely linked to adverse mental health outcomes, increased social isolation, and reduced quality of life among older adults, thereby elevating societal health burdens (3, 4).

Existing academic research on social integration has primarily focused on the social adaptation of general immigrant groups and the social rights of aging migrants (5). Sports, as a form of social culture, have garnered attention for their inherent capacity to foster social integration and group participation, offering a potential pathway for inclusion (6). Despite this promise, a significant gap remains: a bottom-up, logical explanation of how sports facilitate the urban social integration of aging migrants is lacking (7). This gap manifests in two key problems. First, there is a fragmentation of social integration indicators. The process through which sports promote integration is complex, and existing models, many of which lack consensus, cannot be directly applied to this unique group (8). Second, there is an ambiguity regarding the mechanisms of social integration effects. While sports can potentially overcome discrimination, expand social networks, and enhance social capital for aging migrants (9), the precise logical pathways remain vague and underexplored. More importantly, current discussions often treat sports participation merely as a lifestyle choice or a public health metric, largely overlooking its potential as a structured social work intervention. This oversight limits our ability to leverage sports as a professional tool to advance social justice and empower this vulnerable demographic within the framework of sport social work.

Therefore, this study employs grounded theory to address these gaps. We aim to systematically identify the key elements through which public sports promote the social integration of aging migrants and refine the theoretical logic underlying this process. By constructing a coherent model, this research provides evidence-based suggestions for leveraging sports to enhance the social integration of this growing demographic.

This study makes both theoretical and practical contributions. Theoretically, it develops a multidimensional model (logic starting point—logic basis—logic core) based on in-depth interviews and textual data. This model integrates fragmented research findings and systematically reveals the interconnections and logical chain between key elements. Practically, this study extends the value of sports beyond the playing field. It provides actionable evidence for sport social workers and community planners to design precise interventions that go beyond mere physical fitness. By elucidating how sports can reconstruct social support systems and foster identity recognition, this research offers a roadmap for advancing social justice, ensuring that aging migrants (as a marginalized group)can claim their right to the city and achieve lifelong wellbeing through inclusive practice.

## 2 Literature review

The academic discourse on sports and social integration for aging migrants has evolved along two primary trajectories: the theoretical transplantation of social integration frameworks from the general floating population to aging migrants, and the exploration of sports as a specific catalyst for their integration. The following review critically examines these trajectories to identify persistent gaps and situate the novel contribution of the present study.

### 2.1 Research on the social integration of aging migrants

Aging migrants are typically defined as individuals aged 60 and above who leave their familiar environments for reasons such as caring for grandchildren, family reunion, or retirement, relocating to cities where their children reside (10). The conceptual roots of social integration trace back to Durkheim, who employed it as a key variable in explaining societal phenomena like suicide (11). Over time, the focus of integration research shifted toward immigration studies and the aging of migrant populations (12, 13).

Building on sociological, demographic, and psychological theories, scholars have achieved considerable progress in measuring the quality of life, physical and mental health, and social integration of migrant populations, often centering on the questions of “how to integrate” and “the degree of integration” (14–16). For instance, Peilin (17) proposed a hierarchical model of social integration for the floating population, progressing from inner psychological integration to outer systemic integration. Xiao Zirong subsequently synthesized measurement indicators around five dimensions: economic, social, cultural, behavioral, and psychological integration (14, 18). Predominantly, the study of aging migrants has adhered to this broader theoretical framework developed for the general floating population.

However, a critical limitation persists. Aging migrants constitute a distinct social phenomenon within China, characterized by unique motivations and vulnerabilities. The direct application of the general floating population’s integration framework is problematic, as it often fails to capture the nuanced realities of this subgroup (19). A significant shortcoming in prior research is the neglect of heterogeneity among different migrant groups (20). Aging migrants, who embody the dual characteristics of mobility and aging, face a distinct set of challenges. Yet, the specific factors affecting their social integration have not attracted sufficient scholarly attention, resulting in a scarcity of targeted research. While the motivations (e.g., grandparenting) and institutional context (e.g., the hukou system) for aging migrants in China are distinct, the core challenges of integration—such as rebuilding social networks, overcoming cultural and linguistic barriers, and redefining one’s identity and role in later life—resonate with the experiences of older immigrant populations in other national contexts (21, 22). This parallel suggests that while integration frameworks must be context-sensitive, insights can be drawn from comparative perspectives. Consequently, there is a pressing need to identify the unique factors influencing the social integration of aging migrants to supplement and refine the existing theoretical frameworks of population aging and floating population integration.

### 2.2 Research on sports and the social integration of aging migrants

The relationship between sports and immigrant integration has long been a subject of academic inquiry. A central thesis is that sports provide a platform for individuals from diverse backgrounds to connect through shared interests, fostering ability development, expanding social networks, enhancing cohesion, and thereby augmenting group capital (23, 24). A synthesis of the literature reveals two main strands of thought.

The first strand explores the impact of sports on integration. Scholars like Qiu and Jianwei (25) posit that mass sports participation enables aging migrants to find interest-based communities, thereby facilitating social integration. The non-utilitarian, interactive, learning-oriented, and inclusive nature of sports offers a unique mechanism for this process, aligning with the core principle of “people-centered urbanization” (26, 27). The second strand investigates the mechanisms behind this relationship. Sports are viewed as a powerful medium for integration, particularly in cross-cultural settings where they can function as a form of non-verbal communication (28). Proposed mechanisms include strengthening social identity, fostering social connections, promoting cross-cultural understanding, and increasing social capital (29, 30). One model suggests that sports build social networks through interaction, shared interests, habits, and embodied practice, thereby enhancing identity recognition and accelerating integration (7).

While these studies affirm that sports can accelerate integration by expanding social networks (31), they often present an overly optimistic view. The literature frequently overlooks the significant structural and cultural barriers aging migrants face in participating in sports, for which targeted solutions are urgently needed (32). Furthermore, the role of sports is not universally positive. Due to class, linguistic, and economic disparities, sports can sometimes exacerbate hostility and contradictions, creating a field where aging migrants risk further marginalization (33). Indeed, while sports possess the potential to transcend cultural and economic divides, they are not a panacea for integration (34). The proposition that sports directly reconstruct social networks is tenuous, as this process is mediated and constrained by a multitude of practical factors (35, 36).

In summary, a critical analysis of the extant literature reveals three fundamental gaps that this study seeks to address. First, there has been a pronounced oversimplification of the social dimensions of sports and a neglect of the heterogeneity within the aging migrant population. Second, although numerous influencing factors have been proposed, they remain fragmented and lack systematic integration, resulting in an unclear and under-theorized logical pathway. Third, there is a significant disconnect between sociological analysis and professional intervention. Existing studies largely view sports participation as a spontaneous leisure activity, failing to examine it through the lens of Sport Social Work. Consequently, there is a lack of evidence-based frameworks that guide how practitioners can actively leverage sports to advance social justice and promote the lifelong wellbeing of aging migrants as a distinct vulnerable group. This gap is evident both in the China-specific literature and in the broader international scholarship on sport, migration, and aging, which often lacks fine-grained models for this sub-group. This study aims to bridge these theoretical and practical divides. By comprehensively collecting and analyzing data on the “social integration of aging migrants,” supplemented with semi-structured interviews, we employ a rigorous grounded theory approach. This methodology allows us to move beyond theoretical presuppositions, inductively discover key elements, and construct a novel theoretical framework grounded in the Chinese context, yet offering conceptual tools that may inform inquiries in other settings. This thereby significantly expands the breadth and depth of research on the social integration of the floating population.

## 3 Research design

### 3.1 Research method

This study employed grounded theory, a qualitative research methodology designed to generate theory grounded in empirical data. This approach involves the systematic collection and simultaneous analysis of data, through processes of constant comparison, categorization, and conceptualization, to abstract core theories directly from the data (37). Recognized as one of the most rigorous qualitative methodologies, it is particularly suited for investigating emergent social phenomena and complex social processes where existing theories are inadequate (38).

The social integration process of aging migrants is inherently complex and lacks a targeted theoretical framework (8). Consequently, grounded theory was deemed the most appropriate method. It facilitates the collection of rich, process-oriented data, enabling a nuanced exploration of the key elements and theoretical logic through which sports promote social integration. Since its inception in the mid-20th century, grounded theory has evolved into three major schools: the classic approach of Glaser & Strauss, the procedural approach of Strauss & Corbin, and the constructivist approach of Charmaz (39).

This study adopts the Strauss & Corbin procedural approach (40, 41). This choice was driven by the approach’s foundations in symbolic interactionism and pragmatism, which align perfectly with the subject matter. Sports function as a form of symbolic interaction that can expand social networks and facilitate integration (38). The communication and meaning-making that occur within sports settings can be understood as a process of encoding and decoding symbols (30). The Strauss and Corbin approach provides explicit analytical techniques (e.g., the paradigm model) to analyze and verify relationships between categories, which is essential for delineating the complex process under investigation.

**Figure 1:**
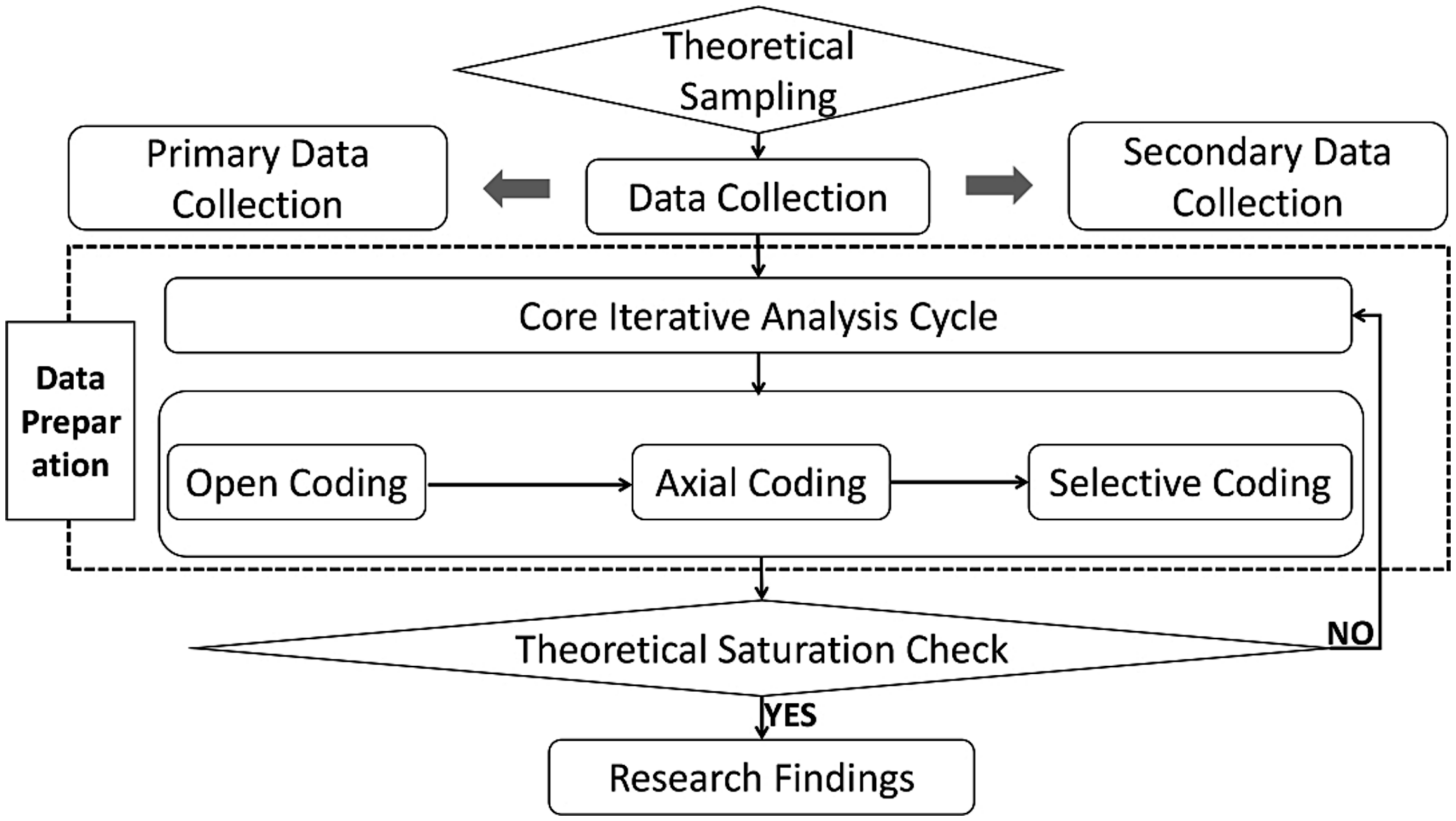
Grounded theory research procedure for model development. Figure illustrates the iterative grounded theory procedure. The process began with theoretical sampling and dual-source data collection (primary interviews, *N* = 34; supplementary texts, *N* = 23). Data underwent a cyclical analysis of open, axial, and selective coding within NVivo, with ongoing analysis guiding further sampling until theoretical saturation was achieved. This rigorous, data-driven process culminated in the construction of the final multidimensional integration model presented in this study.

Following the Strauss and Corbin procedural approach, we engaged in an iterative coding process comprising three core stages: (1) Open coding, (2) axial coding, and (3) selective coding. This process facilitated the abstraction of data from initial concepts to a cohesive theoretical model. The entire analysis was supported by NVivo 12 software to ensure rigor and systematicity in data management. A detailed schematic of the research procedure, illustrating the dynamic interplay between data collection, analysis, and theory development, is provided in Figure 1 (see Section 2.2 for data sources).

### 3.2 Data sources

To ensure theoretical robustness and depth, this study utilized a dual-strategy for data collection, combining in-depth interviews with secondary data analysis.

*Secondary data collection*: We collected 23 supplementary textual materials to achieve theoretical saturation and provide contextual depth. This corpus included: (1) 10 academic papers focusing on aging migrants and social integration in China; (2) 8 policy documents and community service reports from local governments (e.g., aging enterprise development plans, sports facility planning); and (3) 5 news reports and feature articles from mainstream media detailing community activities involving older migrants. These texts were systematically analyzed to inform the interview guide and provide a broader socio-political context for understanding integration mechanisms.

*Primary data collection*: In-depth interviews. Semi-structured interviews served as the primary data source for theory construction. Each interview, lasting between 45 and 90 min, was audio-recorded, transcribed verbatim, and supplemented with field notes on non-verbal cues (42). A follow-up validation check with five randomly selected participants 1 month later confirmed data consistency. In the analysis, the interview transcripts were the principal material for generating codes and categories. The secondary texts were used primarily for contextual understanding, theoretical sensitivity, and triangulation—to challenge, confirm, or elaborate on the emerging concepts from the interviews. The final dataset for grounded theory analysis comprised 34 interview transcripts and 23 supplementary texts, totaling approximately 481,000 words.

*Ethical considerations*: This study received ethical approval from the Institutional Review Board of Yancheng Institute of Technology (Approval No.: YIT-SS-IRB-2024-07-007). All procedures adhered to the Declaration of Helsinki. Prior to interviews, participants received detailed information about the study and provided written informed consent. Participation was voluntary, with the right to withdraw at any time without consequence (Table 1).

**Table 1:** Information related to interview subjects.

No.	Gender	Age	Migration destination	Migration origin
1	Male	62	Jiangsu	Shandong
2	Female	71	Beijing	Henan
3	Male	64	Hunan	Henan
4	Male	63	Tianjin	Henan
5	Male	69	Guangdong	Liaoning
6	Male	72	Guangdong	Liaoning
7	Female	74	Beijing	Shandong
8	Female	65	Beijing	Shandong
9	Female	63	Jiangsu	Hunan
10	Male	69	Shanghai	Hunan
11	Female	66	Shanxi	Sichuan
12	Female	67	Wuhan	Gansu
13	Male	62	Tianjin	Henan
14	Female	61	Chongqing	Henan
15	Female	60	Hebei	Chongqing
16	Female	65	Chongqing	Shandong
17	Female	65	Tianjin	Shandong
18	Male	66	Chongqing	Henan
19	Female	69	Hebei	Shanxi
20	Female	75	Wuhan	Jiangsu
21	Male	74	Tianjin	Shanxi
22	Female	76	Shanghai	Liaoning
23	Female	66	Shanghai	Jiangsu
24	Female	65	Shanghai	Jiangsu
25	Male	64	Shanghai	Shandong
26	Female	68	Shanghai	Shandong
27	Male	69	Chongqing	Shanxi
28	Male	62	Hebei	Jiangsu
29	Female	66	Shanxi	Shandong
30	Female	62	Hebei	Shandong
31	Female	63	Beijing	Shandong
32	Male	73	Beijing	Shandong
33	Female	71	Beijing	Jiangsu
34	Female	72	Beijing	Jiangsu

## 4 Data processing and analysis

The data analysis strictly followed the grounded theory procedures outlined by Glaser (43), comprising three iterative stages of coding: open coding, axial coding, and selective coding. This systematic process ensured that the resulting model was firmly grounded in the raw data.

### 4.1 Open coding

Open coding entails the initial, line-by-line analysis of raw data to identify and conceptualize discrete phenomena (44). We followed a three-step procedure:

(1) *Conceptualization*: We labeled discrete phenomena in the textual data to form initial concepts. For example, the statement “The community doesn’t have spare money to organize sports activities for us every day… they only notify the local people… How can we outsiders know?” was conceptualized as “Community Exclusion.”
(2) *Concept categorization*: Initial concepts were compared and grouped based on semantic similarities into conceptual clusters.
(3) *Abstraction and naming*: Each cluster was abstracted into a higher-level category and given a name reflecting its core characteristic.

This process yielded a systematically coded dataset, the “Record of Social Integration of Aging Migrants.” An excerpt is shown in Table 2.

**Table 2:** Example of record of social integration of aging migrants.

Data coding	Data record	Initial concept
Code 1	The construction of community sports facilities often overlooks the experiences and needs of the aging, creating difficulties for aging migrants in accessing sports services.	Facility limitations
Code 2	Before moving, I frequently participated in sports activities in my community, but after the relocation, due to environmental changes and the adaptation to new interpersonal relationships, my physical exercise has decreased. However, I believe it will gradually return to the previous state.	Environmental discomfort
Code 3	I feel that the sports facilities provided by the community are insufficient, especially for someone like me who enjoys Tai Chi. Now, due to the crowd, I do not particularly enjoy practicing Tai Chi in groups; I prefer running or taking walks because they are less demanding on the venue.	Venue limitations
Code 4	The community also does not have the funds to organize sports activities for us daily; even if they do, they are on a small scale and usually involve local people they are familiar with. How are we, as outsiders, supposed to know about these activities?	Community exclusion
Code 5	Aging migrants in good health are more willing to engage in sports activities, whereas those with economic pressures often devote their time and money to productive labor, neglecting the importance of sports activities.	Economic pressure
Code 6	Existing policies often fail to fully consider the particularities of aging migrants, overlooking their needs in terms of sports services.	Policy Limitations
Code 7	I should exercise more often; at home, there are too many lifestyle differences. Nowadays, young people like to sleep in late, which I cannot stand, and I cannot say much about it. If I say too much, it will lead to arguments. By exercising outside, I avoid seeing these issues and keep my peace.	Family conflict

Due to space limitations, only some coded data records are listed, and the rest are omitted.

Using NVivo 12.0, we applied this procedure to the full dataset. Through iterative comparison and team discussion to ensure reliability, 64 initial concepts were extracted. These were consolidated into 21 initial categories, summarized in Table 3.

**Table 3:** Open coding analysis.

Initial category	Initial concept
Environmental inclusion	Facility limitations, environmental discomfort, venue limitations, community exclusion
Government inclusion	Economic pressure, policy limitations
Family inclusion	Family conflicts, communication difficulties, lack of communication
Intergenerational emotion	Emotional exchange, emotional identification, emotional satisfaction, emotional support
Intergenerational support	Family relationships, family interaction, support from family members
Government support	Government assistance, technical assistance, policy inclusion
Community services	Organizational encouragement, high-quality facilities, community aid
Sport assimilation	Emotional assimilation, shared interests, common topics, behavioral assimilation
Local memory	Nostalgia for hometown, sharing memories, re-creation of hometown, urban–rural interaction
Individual consciousness	Personality differences, exercise awareness, integration awareness, health awareness
Intergenerational consensus	Value consensus, conceptual consensus, family role consensus, consensus on major decisions
Relationship bond	Descendant bonds, interpersonal relationships
Historical memory	Shared memories, past memories
Social memory	Emotional resonance, social recollections, changes in interaction
Sport socialization	Sharing experiences, shared interests, common topics
Individual adjustment	Exercise habits, personal time, habit changes
Individual cognition	Overcoming difficulties, concerns about exercise, cognitive adjustment
Mental health	Depression prevention, self-confidence enhancement, psychological satisfaction
Purpose of sport	Familiarity with the city, killing time
Psychological adaptation	Formation of belonging, resolution of strangerhood
Cultural adaptation	Familiarity with culture, cultural perception, historical learning

The table above provides a sample of the 21 initial categories. The complete set is available upon request.

### 4.2 Axial coding

Axial coding examines relationships between categories. We analyzed, compared, and synthesized the 21 initial categories to group them into more abstract main categories representing broader themes (37). This analysis consolidated the 21 initial categories into 6 main categories, based on their shared properties and theoretical dimensions. Their relationships are detailed in Table 4.

**Table 4:** Axial coding analysis.

Main categories	Initial categories	Connotation explanation
Social inclusion	Environmental inclusion	Establishing an inclusive sports environment (facilities, venues, community) can enhance the sense of psychological belonging among aging migrants.
	Government inclusion	Forming inclusive sports policies from a humanistic perspective can enhance the social identity of aging migrants.
	Family inclusion	In sports situations, an inclusive family life plays an important role in the social integration process of aging migrants.
	Government support	Government support for the physical exercise of aging migrants in terms of policy, funding, and technology.
	Community services	Various sports-related services provided by the community.
Intergenerational relationships	Intergenerational support	Family members provide financial assistance or emotional support for the physical exercise of aging migrants.
	Intergenerational emotions	Emotional communication, understanding, support, and satisfaction from family members regarding the physical exercise of aging migrants.
	Intergenerational consensus	The value consensus or agreement reached between family members and aging migrants in the context of physical exercise.
Social networks	Relationship bonds	Relationship bonds are the foundation of social networks and can help aging migrants establish trust, support, and understanding during sports activities.
	Sports socialization	Sports socialization is the core of the social network and an important means for aging migrants to accumulate emotions and gain a deeper understanding through sports.
	Sports assimilation	Sports socialization is a form of the social network, where aging migrants interact over the long term during sports activities, achieving a transition from heterogeneity to homogeneity.
Social memory	Historical memory	Under the stimulation of sports, aging migrants may recall their past experiences and memories.
	Social memory	The memories formed by aging migrants through social activities generated during sports.
	Local memory	Under the stimulation of sports, aging migrants may recall their local environment and experiences.
Identity recognition	Individual adjustment	Adjustments in behavior, emotions, and thinking of aging migrants after participating in sports.
	Individual cognition	Perception, judgment, and reasoning regarding emotions, sports, and problems after aging migrants participate in sports.
	Individual consciousness	Recognition and perception of exercise, integration, and health among aging migrants after participating in sports.
	Purpose of sports	Recognition and perception of exercise, integration, and health among aging migrants after participating in sports.
Psychological integration	Psychological adaptation	Psychological adaptation is the core of the psychological integration of aging migrants, which involves adapting to the environment after engaging in sports.
	Cultural adaptation	Cultural adaptation is the foundation of the psychological integration of aging migrants, which involves adapting to the culture after engaging in sports.
	Mental health	Mental health is a prerequisite for the psychological integration of aging migrants, and sports can enhance their level of mental health.

### 4.3 Selective coding

Selective coding aims to identify a central, core category that integrates all other categories into a coherent theoretical framework. The core category must be central, frequently appearing in the data, and capable of logically connecting all other main categories (45). We determined that all six main categories were essential. The overarching theme—“the key elements and logical relationships of sports-promoted social integration for aging migrants”—was identified as the core category, integrating all others. The paradigmatic relationships structuring the model are exemplified in Table 5.

**Table 5:** An example of the paradigmatic relationship.

Exemplary relationship structure	Connotation of relationship structure
Social inclusion—sports participation	Inclusive environments, government, and culture are the external protective elements for sports participation.
Intergenerational relationships—sports participation	Intergenerational support, intergenerational emotions, and intergenerational consensus are the internalized elements for sports participation.
Social networks—social integration	Relationship bonds, sports assimilation, and sports socialization, as part of social networks, are the behavioral driving scenarios that accelerate social integration.
Social memory—social integration	Historical memory, social memory, and local memory, as forms of social memory, are the behavioral driving attributions that accelerate social integration.
Identity recognition—social integration	Identity recognition is the ultimate goal for the transformation of the social identity of aging migrants.
Psychological integration—social integration	Psychological integration is the internal motivation for the transformation of the social identity of aging migrants.
Social memory—social networks—social integration	Social memory facilitates social integration through social networks.
Social networks—social memory—social integration	Social networks facilitate social integration through social memory.

### 4.4 Theoretical saturation test

**Figure 2:**
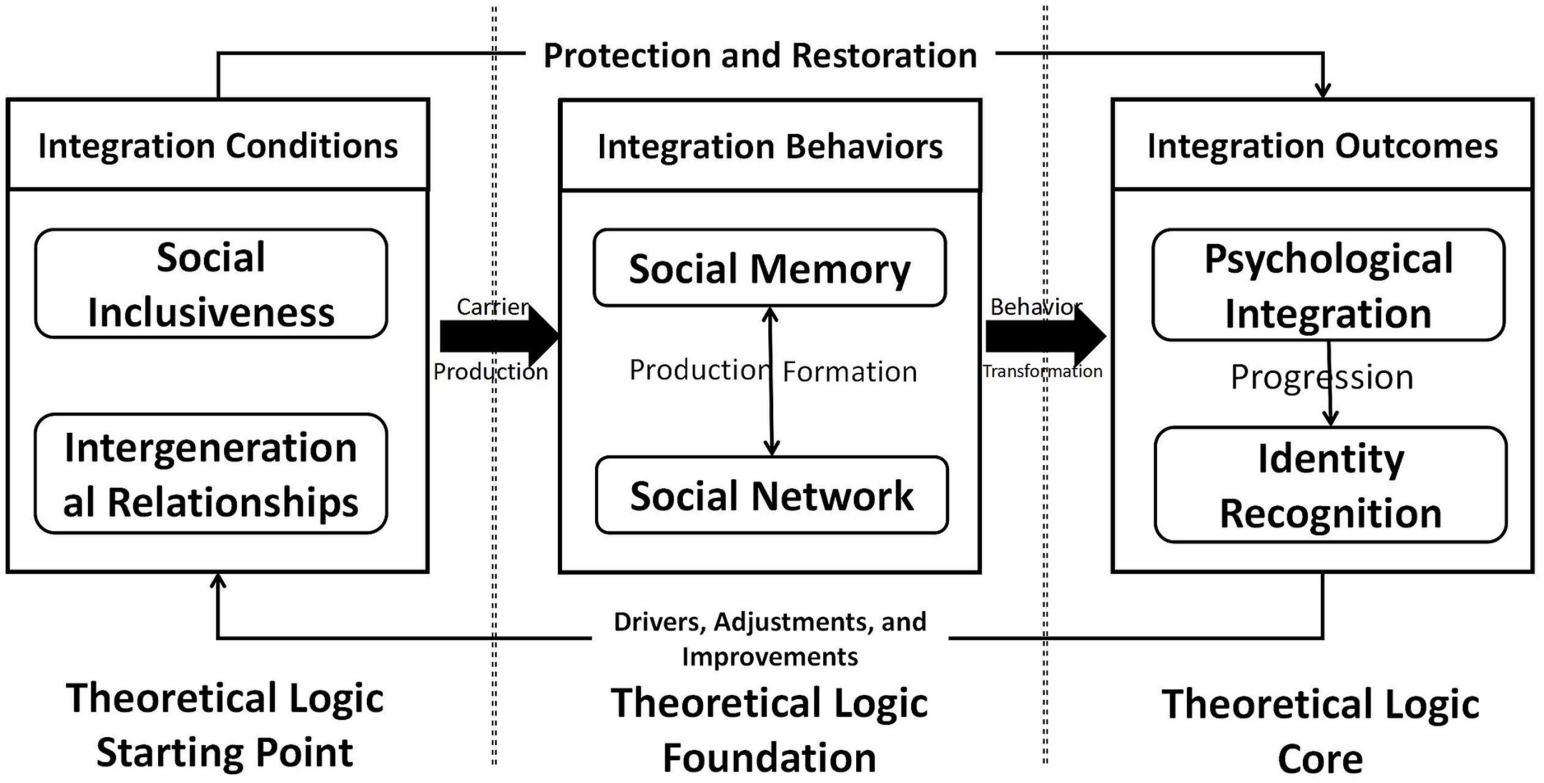
A dynamic, multidimensional model of sports as a catalyst for social integration among aging migrants. This model, derived from grounded theory analysis, illustrates the six core elements and their logical relationships in the integration process. The foundational conditional elements (social inclusiveness, intergenerational relationships) enable sports participation. Participation activates two synergistic behavioral drivers: social networks (structural) and social memory (attributive), which execute the integration process. These behaviors lead to internal outcome elements: psychological integration and, ultimately, identity recognition. Solid arrows denote the primary, facilitative pathway. Crucially, the dashed feedback arrow represents the cyclical progression: achieved outcomes generate new expectations, which recursively place demands on and reshape the initial conditions, making integration a dynamic, adaptive system rather than a linear endpoint.

We conducted a theoretical saturation test (46) using four new interviews and five withheld transcripts. Applying the same coding procedure yielded no new concepts or relationships. Two independent experts in grounded theory and sports sociology reviewed the model and confirmed its coherence and comprehensiveness. This confirmed that theoretical saturation was achieved, as represented in the final model (Figure 2).

## 5 Theoretical elaboration

This section provides a theoretically-grounded elaboration of the six core elements derived from our grounded theory analysis. We move beyond descriptive account to interpret these elements within established sociological and public health frameworks. Our aim is to elucidate the multifaceted and dynamic pathways through which sports facilitate the social integration of aging migrants, thereby addressing the identified research gaps regarding fragmented indicators and ambiguous mechanisms.

### 5.1 Social inclusiveness: the construction and function of the external conditional system

Our findings posit social inclusiveness as a foundational, multi-tiered external conditional system that determines the accessibility and experiential quality of sports participation for aging migrants. This integrated system—encompassing environmental, governmental, and familial dimensions—directly addresses the literature’s noted problem of fragmented integration indicators (8). It demonstrates that successful integration is preconditioned not on isolated factors, but on a holistic architecture of equitable access and psychological welcome, which aligns with the public health imperative of creating enabling environments for healthy aging.

The dimension of environmental inclusiveness—manifested through age-friendly sports facilities, accessible venues, and non-discriminatory community spaces—forms the most immediate layer. It is the tangible interface where initial belonging is negotiated. Participant S17’s account epitomizes this: “The community’s sports facilities are simple to operate… While exercising, one can also chat with neighbors. Initially, they were afraid of being ridiculed for their accent, but later found the locals very enthusiastic.” Such an environment reduces initial barriers and fosters a sense of safety and belonging, which are fundamental psycho-social prerequisites for sustained participation and mental wellbeing. However, this community-level acceptance often requires macro-level scaffolding to be sustained and scaled. This is where governmental inclusiveness becomes critical. It provides legitimacy, stability, and resources through inclusive policies, targeted funding, and technical assistance. The absence of such support can render community efforts ineffective. Governmental action, therefore, functions to actively eliminate structural barriers and signal institutional recognition, addressing aging migrants not merely as a familial responsibility but as citizens with equal rights to recreational and social resources—a core principle of health equity.

Furthermore, our model introduces family inclusion as a crucial, yet often overlooked, component of this external system. For many aging migrants, the family is the primary reason for migration but can also become a site of tension and constraint. Sports can offer a vital respite and a space for autonomy. Participant S13 noted using exercise to avoid familial disagreements: “By exercising outside, I avoid seeing these issues and keep my peace.” Therefore, an inclusive family environment that understands, respects, and supports the older member’s pursuit of sports participation is an internalized external condition—a critical psycho-social support that directly influences the individual’s capacity to engage with wider society. The lack of such a multi-scalar inclusive system constitutes a form of compounded exclusion, denying aging migrants full participation in the social and recreational life of the city, which are key social determinants of health. Thus, building this inclusive sports ecosystem is a concrete public health strategy, operationalizing the “active aging” framework by shifting the focus from treating deficits to proactively creating the social and physical conditions that promote health, social connection, and wellbeing as a matter of right. This aligns with the macro-practice goal of sport social work, where practitioners must act not only as service providers but as policy advocates to dismantle these structural barriers and advance spatial justice.

### 5.2 Intergenerational relationships: the activation and transformation of the internal kinetic mechanism

Within the private sphere of the family, intergenerational relationships emerge not as a static background but as a dynamic internal kinetic mechanism. This insight addresses a significant gap in the literature which has neglected the heterogeneity and internal micro-dynamics of migrant groups (20). For aging migrants, the family is both the primary pull factor and the initial social universe in the new city; its health and functionality are thus paramount to their own psycho-social health and integration capacity. Our model reveals a transformative virtuous cycle activated by sports: Support → Emotion → Consensus.

The first link, intergenerational support, involves practical (e.g., financial, informational, childcare) and emotional encouragement from adult children for their parents’ sports activities. This support acts as a crucial enabler, reducing logistical barriers and providing the initial “permission” and motivation for engagement. As Participant S27 indicated, shared family activities promote communication. This relational support is foundational, echoing public health understandings of family support as a protective factor against isolation and depression.

This relational dimension feeds directly into the second link: the cultivation of positive intergenerational emotions. Shared sports activities, often centered on non-verbal, embodied interaction, create a unique and neutral platform outside the potential conflicts of daily domestic life. This shared experience fosters mutual understanding, enjoyment, and emotional identification, thereby directly countering feelings of loneliness and anxiety commonly reported among this population (19). The case of S24 is illustrative: “Sometimes they hardly talk to their son in a day. In order to exercise, they joined a mountain climbing club, and their son, who was interested, would accompany them… their father-son relationship improved.” Here, sports provided the shared, positive context for re-establishing an emotional connection that routine life had eroded, enhancing relational quality and emotional wellbeing.

The culmination of this process is the formation of intergenerational consensus. The shared emotional foundation built through sports facilitates alignment on values, lifestyles, and roles within the new life context. This consensus is not about uniformity but about achieving mutual respect and understanding. S11’s experience highlighted how sports create a “unique” basis for such understanding. Importantly, this consensus has a recursive effect, reinforcing and deepening the initial intergenerational support, thereby creating a self-reinforcing, positive feedback loop.

Theoretical engagement with Family Systems Theory enriches our interpretation. Sports participation by the aging migrant can be seen as a positive perturbation that alters the family homeostasis. It introduces a new, health-promoting activity that can recalibrate roles, improve communication patterns, and strengthen emotional bonds. Therefore, sports act as a catalytic family health intervention, transforming the family system from a potential source of stress into a powerful internal driver of integration and resilience. By detailing this internal kinetic mechanism, our study provides a nuanced explanation for how family relational health, when positively activated through shared activities like sports, becomes a critical determinant of successful social integration and, by extension, mental and emotional wellbeing for aging migrants. Consequently, this internal mechanism offers a strategic entry point for family-centered sport social work, utilizing sports as a therapeutic medium to repair intergenerational ruptures.

### 5.3 Social networks: the behavioral-driven scenario from “node” to “Nexus”

Social networks represent the behavioral-driven structural scenario where the potential for integration materializes into concrete social action and relational change. This conceptualization clarifies our model: social networks are the evolving architecture of relationships. Prior to migration, aging migrants are typically embedded in dense, multiplex networks (“line-to-line” ties). Upon moving, they often become isolated “nodes,” experiencing a profound rupture that leads to displacement and exclusion (47)—a major risk factor for poor mental health and accelerated decline. Sports function as a powerful engine for reconstructing these networks through a discernible three-stage process, each stage contributing to the accumulation of social capital, a well-established social determinant of health.

Stage 1: Forging new relationship ties. Sports activities, especially group-based ones, provide structured, low-pressure opportunities for initial contact. The shared focus lowers interaction barriers. Interviews revealed that the sense of being cared for in these groups significantly enhanced belonging. Participant S28 mentioned mutual aid within a square dance WeChat group: “We look after each other’s stuff, even help watch grandchildren sometimes.” These nascent ties are the first crucial strands in weaving a new social safety net, providing not only practical help but also initial perceived social support, buffering against stress.

Stage 2: Facilitating sports socializing. This stage involves a deepening of interactions, where relationships transition from being activity-specific (“sport buddies”) to more generalized friends. Participant S36’s experience with a cycling group that arranged regular rides and maintained a WeChat group for social coordination is a prime example. This stage is core to the development of “bridging” social capital (21, 22)—connections to a broader, more diverse set of people beyond the family. It transforms the aging migrant from an isolated node into an active participant in the social fabric of the city, directly combating social isolation.

Stage 3: Leading to sports assimilation. Here, “assimilation” is used in a sociological sense to denote a process of cultural learning and adaptation (48). Through long-term, embodied co-practice in sports-based networks, aging migrants begin to internalize local norms, values, and rhythms. As Participant S46 alluded, this requires personal adjustment. This is not a loss of original identity but an expansion—a process of becoming culturally fluent and “at home” in the new city. This deep socio-cultural embeddedness is a key outcome for sustainable integration and long-term psychological adjustment.

*Clarification of conceptual role*: This detailed pathway addresses the ambiguity in the literature regarding mechanisms (7, 9). We specify that social networks are the dynamic relational structure that is actively built and expanded through sports. They are the scenario or the vehicle for interaction. The content and meaning flowing through these networks—shared histories, values, emotional support—will be addressed in the next section on social memory. This analytical distinction is crucial: networks provide the structural opportunity for health-promoting social connection, while the quality and meaning of those connections influence the psycho-social benefits derived. Public health interventions aiming to leverage sports for integration must, therefore, focus on both creating opportunities for network formation (structural intervention) and fostering positive, meaningful interactions within them (attributive intervention).

### 5.4 Social memory: the behavioral-driven attribution of meaning-making and reconstruction

While social networks provide the essential structural scaffolding for interaction, social memory constitutes the behavioral-driven attributive process that fills this structure with personal and collective meaning, transforming mere contact into psychologically resonant belonging. The concept of social memory—understood as the process by which collective experiences, knowledge, and cultural patterns are encoded, stored, and recalled within a social context (49)—provides a novel theoretical lens largely absent from literature on sports and migrant integration. This focus on meaning-making addresses a key public health concern: social connection without meaningful engagement may not suffice to combat deep-seated loneliness or foster a true sense of belonging, which are critical for mental health.

Upon migration, aging migrants carry a rich tapestry of historical and local memories—ingrained ways of life and social scripts. This “memory baggage” can violently clash with the new urban environment, leading to cognitive dissonance and cultural disorientation, exacerbating feelings of alienation. Participant S34’s statement captures this starkly: “the urban environment is good, but life is really not used to it. In the past, in the hometown… everyone used each other’s things. Now no one knows each other, and they can’t open their mouths.” The original social memory becomes dysfunctional, creating a void.

Our model reveals sports as a unique mnemonic field offering dual pathways for managing this disruption:

(1) *The reconstructive pathway*: Physical activity acts as a catalyst for reconstituting historical memory in a bridge-building manner. The non-competitive, socially-oriented nature of activities like group dancing or walking fosters conversation, turning the sports field into what Nora terms a lieu de mémoire (site of memory). Participant S56’s experience is exemplary: discovering shared military experiences with a “ball friend” led to the feeling that “he and I have been old friends for many years.” Here, shared exertion creates a safe space for exchanging personal histories, allowing the past to be re-narrated and validated within a new social context, thus weaving threads of personal history into the present social fabric. This process mitigates the trauma of abrupt cultural loss.

(2) *The formative pathway*: Concurrently, sports arenas become active sites for generating new, locally-grounded collective memories. As Arendt theorized, full human existence requires a public sphere of shared action (50). For aging migrants, sports open this “shared field with others.” The repeated, shared experiences of morning Tai Chi or cycling trips with new companions create a reservoir of common references and joint histories. This process, as S55 noted, “makes up for the emotional deficiency” caused by ruptured old ties. Following Halbwachs (49), individual memory is sustained by social frameworks; here, the new social framework of the sports group scaffolds the formation of new, positive memories associated with the host city, which are fundamental for psychological attachment.

Conceptual distinction and synergy: It is crucial to distinguish social memory from social networks. Social networks are the structural channels or the relational circuit through which interaction flows. Social memory is the meaningful content, the cognitive-emotional software (shared stories, nostalgia, new jokes, common routines) that runs through those channels and gives them emotional depth and significance. They are analytically distinct but empirically synergistic: networks circulate memory, and memory enriches and solidifies networks. This synergy explains how integration moves beyond superficial contact to deep, psychological acceptance.

*Adding theoretical nuance*: Memory conflict and negotiation. The process is not solely harmonious. Data hints at instances where an aging migrant’s “local memory” (e.g., of informal, ritualized village exercise) clashed with the host city’s more standardized, institutionalized sports culture. These minor conflicts, however, occur within the low-stakes, flexible arena of sports, becoming points of practical negotiation and adaptive fusion, rather than irreconcilable difference. This negotiation itself becomes part of the new collective memory, enriching the integrative process.

### 5.5 Psychological integration: the internal metamorphosis from the “Burden of Freedom” to active engagement

Psychological integration is the pivotal internal outcome element, representing the internalization of external social gains into a stable subjective state of belonging. It directly determines whether an aging migrant feels integrated, which is the ultimate mediator between social activity and mental health outcomes such as reduced depression and anxiety. Our analysis uncovers a profound paradox using Fromm’s framework (51): post-migration “freedom” from old social structures can precipitate a crushing sense of loneliness and irrelevance—the “burden of freedom.” Participant S40 described this vividly: no longer the family’s main labor force and having lost original social ties, they felt an “unprecedentedly strong” sense of loneliness despite having more free time. This “burden” stems from the anxiety of constructing a meaningful life ex nihilo, a significant psycho-social stressor.

Within this context, sports emerge not as a mere pastime but as a structured and positive form of “escape from freedom.” Fromm posited that individuals may seek to escape the burdens of isolated selfhood by merging with new communities or authorities (52). Sports represent a healthy, constructive escape mechanism. It is a voluntary submission to a new structure—the schedule of a walking group, the rules of a game. This structure replaces the oppressive void of unstructured time with a purposeful “second career,” actively combatting loneliness. Participant S22 stated, “I used to feel that the time in the morning was very long, but now it’s much better to come out and exercise… than staying at home,” and S57’s diary-like account of a sports-filled day illustrates this replacement of lost “primary constraints” with new, chosen “secondary ties.” This directly addresses social isolation, a key public health risk factor.

Beyond alleviating loneliness, sports facilitate deeper embodied cultural adaptation. Regularly navigating public sports spaces allows for an intuitive, felt understanding of the city’s culture—a form of experiential learning more profound than passive observation. Participant S29 noted that through cycling, they made local friends and participated in cultural center activities, fostering a sense of inclusion prior to any formal identity shift. This underscores a critical sequence: psychological integration (feeling at home) is the necessary affective groundwork for stable identity reconstruction (knowing oneself as belonging). By providing a mechanism to overcome the “burden of freedom,” sports directly promote psychological resilience and adaptive capacity, which are core components of mental health in later life and successful aging.

### 5.6 Identity recognition: the ultimate outcome of self-reconstruction and embodied agency

Identity recognition constitutes the ultimate outcome of the integration process, wherein aging migrants develop a renewed, positive, and agentic understanding of their selfhood within the urban context. Constrained by dual marginality, they often struggle to derive a sense of value beyond dependent familial roles. Sports provide a critical domain for positive identity reconstruction through embodied agency, which is intrinsically linked to self-esteem, purpose in life, and overall psychological wellbeing—key metrics in public health and gerontology.

This reconstruction operates via two interconnected pathways:

(1) *Pathway of positive bodily feedback*: Engaging in physical activity generates direct, tangible evidence of capability and vitality. The sensations of improved strength, endurance, and mood (53) actively counter the narrative of decline associated with aging and the passivity that can accompany migration. As central to embodiment theory, the body is the primary medium through which we experience and constitute our self (54). When S25 stated, “more exercise can lead to a longer life,” it reflects a cognitive shift: through their own agency (exercise), they are actively shaping their health and future. This sense of bodily mastery and self-efficacy forms the foundational, somatic layer of a new, empowered identity.
(2) *Pathway of role-based identity conferral*: More powerfully, sports can create specific, valued social roles distinct from familial ones. The case of S33, who assumed the identity of a “Tai Chi coach,” is paradigmatic. This role conferred status, authority (“discourse power”), and a clear sense of purpose. They transitioned from a potentially peripheral family figure to a leadership figure and central node in a community network. This exemplifies the acquisition of a powerful “role-based identity” (55), earned through demonstrated skill and commitment, not merely ascribed by age or kinship.

*Theoretical and public health synthesis*: Engaging with Social Identity Theory, the sports team or club becomes a salient new “in-group,” providing a potent source of social identity and self-esteem. Reinforcement from this group and from family (“My children praise me for being so active”) further solidifies this new self-conception. Ultimately, sports help answer the existential question “Who am I here?” with powerful, multifaceted answers: a healthy individual, a skilled coach, a valued teammate, an active local. This transition from a passive, ascribed identity (“grandparent from elsewhere”) to an active, achieved identity is not only the culmination of social integration but also represents a significant gain in psycho-social resources, directly contributing to successful and healthy aging. From a social work perspective, this transition represents a critical process of empowerment, where the vulnerable group reclaims agency and resists marginalization through embodied practice.

### 5.7 The theoretical logic process: a dynamic system of integration with cyclical progression

The six core elements are interlinked within a dynamic, systemic process. This section synthesizes them into a coherent theoretical logic, delineating a pathway characterized not by linearity but by dynamic cyclical progression. This feedback-driven model accounts for the evolving, lifelong nature of integration, offering a robust framework for understanding long-term adaptive success and wellbeing in later-life migration.

Phase 1: The Formation of Foundational Conditions. The process is preconditioned on the synergistic establishment of social inclusiveness (the external conditional system) and intergenerational relationships (the internal kinetic mechanism). These elements collectively determine the initial “capacity to participate.” An inclusive environment lowers structural and psychological barriers, while supportive family relationships provide the necessary internal motivation and security. The absence of either can curtail participation at its inception, highlighting that integration is a multi-level process requiring both supportive policies and functional family dynamics.

Phase 2: The Synergistic Execution of Integration Behaviors. Once participation is initiated, the core engine of the process engages: the synergy between social networks (the structural-behavioral driver) and social memory (the attributive-meaning driver). Sports create a common “scenario” for interaction (56). Within it, new networks are constructed (forging ties → socializing → cultural assimilation), providing the relational structure. Concurrently, the arena acts as a mnemonic field where memory is actively managed: historical memory is reconstructed to bridge the past, and new local memory is formed to anchor the present. Crucially, these two drivers are mutually constitutive: networks provide the channels for memory to circulate and be shared, while social memory provides the meaningful content and emotional resonance that deepen, stabilize, and give purpose to network ties. This synergy is the active mechanism that pushes the individual toward deeper cultural and psychological adaptation.

Phase 3: The Internalization of Outcomes and Cyclical Recalibration. The behavioral execution directly catalyzes the internalization of outcomes. Successfully navigating new networks and accumulating shared memories enables individuals to overcome the “burden of freedom,” leading to psychological integration—a sense of belonging and reduced loneliness. This internal, affective state then enables identity recognition, where through bodily agency and new social roles, a positive, renewed self-concept is formed.

The Critical Feedback Loop: The model’s explanatory power lies in its cyclical correction mechanism. The integration conditions have a continuous, protective effect on outcomes. More importantly, the achieved outcomes recursively influence the initial conditions. A newfound identity (e.g., as a “local sports enthusiast”) generates “higher-level expectations” (38) (e.g., for better facilities, more advanced activities). These expectations place new demands on both the external system (social inclusiveness needs to provide more sophisticated resources) and the family dynamic (intergenerational relationships may need to adapt to support this new, more active identity). This triggers a new, more advanced cycle of negotiation, participation, and integration. Therefore, our model presents social integration not as a finite endpoint but as a self-correcting, progressively complexifying adaptive system. This dynamic view is essential for public health planning, suggesting that interventions must be ongoing and responsive to the evolving needs and identities of aging migrants, supporting them through successive cycles of integration and growth.

### 5.8 Implications for sport social work practice: from logic to action

While this grounded theory model elucidates the mechanism of integration, its applied value lies in serving as an evidence-based framework for Sport Social Work (SSW). To move “beyond the playing field” and advance social justice, this study proposes that sports participation should be reconceptualized as a structured social work intervention.

Guided by the model’s logic, we identify three dimensions for professional SSW practice:

*Micro-level*: Empowerment and Identity Reconstruction. Aligning with the Identity Recognition element (4.6), practitioners should adopt a strengths-based perspective. Instead of viewing aging migrants solely as service recipients, social workers can design programs that appoint them as peer coaches or team leaders. This strategy fosters role-based identity, transforming sports from a leisure activity into a vehicle for empowerment, directly countering the “burden of freedom” and restoring psychological agency.

*Mezzo-level*: Facilitating Network Weaving. The model highlights Intergenerational Relationships and Social Networks as kinetic drivers. SSW interventions should focus on network weaving rather than just organizing games. Practitioners can act as mediators in intergenerational sports programs to resolve family conflicts, and actively integrate elements of Social Memory (e.g., traditional hometown sports) to create cultural safety zones. This helps reconstruct the fractured social support system of migrants.

*Macro-level*: Advocacy for Spatial Justice. Grounded in the Social Inclusiveness dimension (4.1), sport social workers must assume the role of advocates. The exclusion of aging migrants from urban spaces is a social justice issue. Practitioners should leverage the evidence from this model to lobby for inclusive resource allocation, ensuring that this vulnerable demographic exercises their right to the city through equitable access to public sports amenities.

## 6 Conclusion

### 6.1 Research findings and theoretical contribution

This study employed a rigorous grounded theory methodology to develop a novel multidimensional model that elucidates how sports catalyze the social integration of aging migrants in urban China. Our findings affirm the irreplaceable role of sports as a community-based public health intervention, functioning through a dynamic system of six interconnected elements.

The primary theoretical contributions are threefold. First, the model identifies and conceptualizes six core elements, organized within a clear logic: social inclusiveness (external condition) and intergenerational relationships (internal condition) form the foundational capacity for participation; social networks (behavioral-structural driver) and social memory (behavioral-attributive driver) synergistically execute the integration process; and psychological integration and identity recognition (outcome elements) mark the successful internalization of belonging. The explicit theorization of intergenerational relationships as an internal kinetic mechanism and social memory as a meaning-making driver addresses prior neglect of this group’s heterogeneity and the fragmentation of integration indicators.

Second, the study reveals these elements are interdependent within a dynamic, cyclical system. They form a synergistic chain wherein conditions enable behavioral drivers, which produce psychological and identity outcomes. This systemic view explains the process of integration, resolving ambiguity about its mechanisms.

Third, and most significantly, the research delineates the complete theoretical logic of this process, characterized by a feedback loop. The model illustrates how foundational conditions foster participation, which activates the synergistic drivers of networks and memory, leading to psychological and identity outcomes. Crucially, achieved outcomes generate “higher-level expectations,” which recursively place new demands on and recalibrate the initial conditions, initiating a new cycle of integration. This conceptualizes social integration not as a finite goal but as an ongoing, adaptive, and self-correcting process—a perspective crucial for long-term public health strategies aimed at sustainable wellbeing. Crucially, explicitly aligning with the special issue’s focus, this dynamic model provides a theoretical grounding for Sport Social Work. It demonstrates how sports can be operationalized to reconstruct social support systems, thereby extending the discipline’s reach beyond the playing field to address the complex integration challenges of vulnerable populations.

### 6.2 Implications for sport social work and public health practice

The model offers concrete, actionable insights for policymakers and community planners seeking to leverage sports for health promotion and social inclusion.

*Designing intergenerationally-inclusive sports programs*: Given the critical role of family as an internal kinetic mechanism, community sports initiatives should explicitly encourage multi-generational participation. Examples include “Family Sports Days,” grandparent-grandchild fitness courses, or intergenerational walking clubs. Such programs structurally facilitate the “Support → Emotion → Consensus” cycle within families, turning the family unit into an active partner in the integration process.

*Professionalizing sport social work interventions*: There is an urgent need to empower community staff not merely as fitness instructors, but as sport social workers. Training curricula should prioritize cultural competency and intergenerational mediation. Practitioners must be equipped to utilize sports settings as a ‘non-clinical’ environment to build rapport, identify signs of social isolation, and link aging migrants to broader social welfare resources, thereby integrating physical health with holistic social care.

*Leveraging sports for mental health promotion*: Public health campaigns should frame community sports participation not only as physical activity but as a validated strategy to combat social isolation and loneliness among aging migrants. Partnerships between public health departments, community sports centers, and mental health services can facilitate referrals and create integrated psycho-social support pathways.

### 6.3 Transnational relevance and boundary conditions

While grounded in the specific context of Chinese aging migrants (e.g., influenced by the hukou system and familialist culture), the core mechanisms of the model possess significant transnational relevance for public health research. The dual drivers of social networks and social memory, the internal role of family dynamics, and the progression from psychological adaptation to identity reconstruction are likely universal psychosocial processes in migrant integration. However, their relative weight and manifestation will vary. For instance, in societies with stronger formal welfare systems, the role of governmental inclusiveness may differ, while in more individualistic cultures, the function of non-familial social networks might be even more pronounced. Thus, the model serves as a heuristic framework for comparative research. Its value lies not in offering a one-size-fits-all prescription, but in providing a structured set of relational and processual concepts to guide context-sensitive investigations and interventions in other countries facing similar challenges of aging and migration.

### 6.4 Limitations and future directions

This study has limitations that chart a course for future research. The cross-sectional design, while suitable for model building, limits causal claims. Future longitudinal or panel studies are needed to trace the dynamic, non-linear progression of integration over time. While the qualitative approach uncovered depth, future work could develop and validate a quantitative scale based on our six elements to measure the integration process. Additionally, research could employ the Delphi method with interdisciplinary experts (in sociology, public health, sports policy) to further refine and prioritize the model’s elements for policy action.

Notwithstanding these limitations, this study provides a robust theoretical framework that advances academic understanding and offers a public health-informed roadmap for fostering inclusive, cohesive, and healthy aging societies roadmap for fostering inclusive, cohesive, and healthy aging societies. Ultimately, this research asserts that sport social work is indispensable in ensuring that aging migrants can claim their right to the city and achieve social justice through the strategic power of sports.

## Data Availability

The raw data supporting the conclusions of this article will be made available by the authors, without undue reservation.
